# Odor induced functional connectivity alteration of POC-anterior frontal cortex-medial temporal cortex in patients with mild cognitive impairment

**DOI:** 10.3389/fnagi.2025.1502171

**Published:** 2025-02-20

**Authors:** Weiping Li, Jianan Zhou, Shuying Li, Min Wu, Yajing Zhu, Qian Chen, Futao Chen, Xuefeng Ma, Xin Zhang, Zhengge Wang, Jiaming Lu, Bing Zhang

**Affiliations:** ^1^Department of Radiology, Nanjing Drum Tower Hospital Clinical College of Nanjing University of Chinese Medicine, Nanjing, China; ^2^Department of Radiology, Nanjing Drum Tower Hospital Clinical College of Nanjing Medical University, Nanjing, China; ^3^Department of Radiology, Nanjing Drum Tower Hospital, Affiliated Hospital of Medical School, Nanjing University, Nanjing, China; ^4^State Key Laboratory of Pharmaceutical Biotechnology, Nanjing University, Nanjing, Jiangsu, China; ^5^Institute of Medical Imaging and Artificial Intelligence, Nanjing University, Nanjing, China; ^6^Medical Imaging Center, Department of Radiology, Nanjing Drum Tower Hospital, Affiliated Hospital of Medical School, Nanjing University, Nanjing, China; ^7^Jiangsu Key Laboratory of Molecular Medicine, Nanjing, China; ^8^Institute of Brain Science, Nanjing University, Nanjing, China

**Keywords:** mild cognitive impairment, olfactory fMRI, POC, anterior frontal cortex, medial temporal cortex, gPPI

## Abstract

**Background:**

Mild cognitive impairment (MCI) is associated with an increased risk of dementia in older adults. Olfactory impairment may indicate prodromal dementia, yet its underlying mechanisms are not fully understood. This study aimed to investigate the alterations in functional connectivity (FC) of odor-induced olfactory neural circuits in MCI patients.

**Methods:**

The study included 39 MCI patients and 42 normal controls (NCs). All subjects underwent cognitive assessments, olfactory behavior tests, and odor-based functional magnetic resonance imaging (fMRI). Differences in FC within olfactory circuits were analyzed using the generalized psychophysiological interaction (gPPI) method.

**Results:**

Mild cognitive impairment patients showed significant cognitive deficits, including lower scores on the Mini-Mental State Examination (MMSE) and Montreal Cognitive Assessment (MoCA), alongside impairments in episodic memory, visuospatial memory, executive function, language, attention, olfactory threshold, and total olfactory function. Compared to NCs, MCI patients exhibited reduced activation in the bilateral primary olfactory cortex (bPOC) during olfactory stimulation. Odor-induced bPOC activation correlated with olfactory thresholds across the cohort. During odor stimulation, MCI patients showed increased FC from the bPOC to the right anterior frontal lobe, particularly the middle frontal gyrus (MFG) and superior frontal gyrus (SFG). Conversely, FC from the right anterior frontal lobe to the medial temporal cortex, including the fusiform and parahippocampal gyri, was reduced in MCI patients. Increased FC from the bPOC to the right SFG/MFG negatively correlated with episodic memory, while decreased FC to the right fusiform/parahippocampal gyri positively correlated with attention, language ability, and olfactory identification.

**Conclusion:**

This study indicates that impaired FC within the primary olfactory cortex (POC)-anterior frontal cortex-medial temporal cortex circuit is a sensitive neuroimaging marker for early MCI identification. The primary dysfunction appears in the POC, suggesting that FC alterations from this region may provide novel diagnostic and therapeutic avenues for early intervention.

## Introduction

Alzheimer’s disease (AD), the leading cause of dementia, is rapidly becoming one of the most expensive, lethal, and burdensome diseases of this century (Scheltens et al., 2021). Early risk screening, diagnosis, and intervention during the preclinical stage have become focal points in the prevention and management of dementia (Janssen et al., 2022). Mild cognitive impairment (MCI) is an intermediate condition between normal aging and AD, representing a critical window for early detection and intervention. The prevalence of MCI among older adults is 6.7% in the 60–64 age group, rising to 25.2% in those aged 80–84 years (Petersen et al., 2018). Importantly, some MCI patients, with timely intervention and effective treatment, can revert to cognitively normal status or stabilize without further deterioration (Walsh et al., 2020). Therefore, early screening and intervention for the MCI population are essential for preventing further cognitive decline and improving outcomes.

Olfactory dysfunction, detectable in AD and MCI, has emerged as a promising early biomarker for improving the diagnosis of AD (Dan et al., 2021; Jesmanas et al., 2021; Jobin et al., 2021b). In cognitively healthy older adults, reduced baseline olfactory function is associated with an increased risk of cognitive decline, higher conversion rates to MCI, and a greater likelihood of developing dementia (Pacyna et al., 2023; Yaffe et al., 2017). Most behavioral studies have found significant reductions in olfactory detection and identification abilities in patients with MCI when compared to age-matched healthy controls, regardless of the specific olfactory test employed (Liu et al., 2024). Decreased olfactory function was correlated with reduced volumes in several brain regions, including the temporal lobe (comprising the hippocampus, parahippocampal gyrus, and fusiform gyrus), olfactory-related areas (such as the piriform cortex, amygdala, and entorhinal cortex), as well as the precentral and postcentral gyri and the globus pallidus (Bothwell et al., 2023). Importantly, olfactory training has been shown to increase the volume of olfactory-related brain regions, such as the olfactory bulb (OB) and hippocampus, while also altering functional connectivity (FC) (Al Aïn et al., 2019; Vance et al., 2024). Among older adults living in the community, impairment in olfactory function was found to be a better predictor of cognitive decline than deficits in verbal episodic memory (Devanand et al., 2015). Additionally, poorer olfactory function predicted the onset of MCI and was associated with both global and local Aß deposition (Tian et al., 2022). Studies have also indicated that higher olfactory scores are linked to a lower risk of transitioning from unimpaired cognition to MCI, and from MCI to dementia (Knight et al., 2023). Research has demonstrated that the pathological process begins with neurotic plaques and neurofibrillary tangles in the entorhinal cortex, progressively extending to other areas of the temporal cortex and neocortex in MCI patients (Ubeda-Bañon et al., 2020). Additionally, the extent of tau deposition within the entorhinal cortex has been linked to impaired memory performance in cognitively normal individuals (Lowe et al., 2019). This sequence of neurodegenerative changes, particularly in regions involved in olfactory processing, explains why olfactory decline is often one of the earliest detectable clinical markers of MCI, occurring when individuals are still cognitively normal. This highlights the potential of olfactory dysfunction as an early diagnostic indicator, providing an opportunity for earlier intervention and monitoring in individuals at risk of progressing to AD.

Olfactory signals are relayed from the olfactory neurons in the nose to various brain regions through the OB. Interneurons establish connections with the primary olfactory cortex (POC) and the anterior hippocampus, contributing to the processes of smell recognition and memory retrieval (Kharlamova et al., 2023). This region is notably among the initial sites impacted by neurodegenerative alterations, including tau pathology and β-amyloid deposition, both of which are hallmarks of AD. The hippocampus is essential for olfactory working memory, while odor identification engages Broca’s area, as well as the orbitofrontal and prefrontal cortices, which are also key components of the olfactory cortical network. The frontal cortex extensively projects to subcortical regions such as the hippocampus, amygdala, and hypothalamus, which are vital for memory processes (Friedman and Robbins, 2022). It integrates complex perceptual information received from the temporal cortices and other associative areas to facilitate cognitive functions (Bhattarai et al., 2022). Previous research has identified particular brain regions associated with olfactory deficits in aging, including the POC within the piriform cortex, the amygdala, and the anterior olfactory nucleus (Attems et al., 2015). Subject cognitive decline (SCD) subjects exhibited abnormal FC of the olfactory neural circuits (POC-hippocampus-frontal cortex neural circuits) during odor stimulation. However, the volume of the POC did not show any abnormalities (Zhang et al., 2024). Numerous studies have also shown a decrease in both the activated volume observed during task-based functional magnetic resonance imaging (fMRI) and the structural volume of the POC in patients with AD and MCI (Vasavada et al., 2015; Zhang et al., 2019). Evidence has emerged linking olfactory impairment to MCI in older adults (Cieri et al., 2024). Nevertheless, the mechanisms driving this association are not well elucidated, particularly regarding the FC between the POC and advanced olfactory regions during odor stimulation in MCI.

In this study, we conducted an odor-induced fMRI paradigm to evaluate the alterations of the neural circuits and cognitive functions in patients with MCI. The results are expected to reveal the olfactory FC alterations in MCI and provide clues to the underlying mechanisms that connect olfactory neural circuits with mild cognition decline. Further, it can provide valuable insights into changes in olfactory FC as sensitive imaging markers, presenting a non-invasive method for identifying individuals at high risk during the early stages of AD.

## Materials and methods

### Participants

A total of 81 participants, comprising 39 MCI patients and 42 normal controls (NCs), who met the inclusion criteria, were enrolled in the study in Nanjing Drum Tower Hospital. All participants were right-handed and underwent neuropsychological tests, olfactory behavior measurements, and olfactory MRI scanning.

Inclusion criteria were age 55–80, right-handedness, and a minimum education level of 8 years. Exclusion criteria included a history of cerebrovascular disease, severe depression and anxiety, other neurological conditions contributing to cognitive decline, MRI contraindications, and disorders affecting olfactory function. Patients with MCI met the clinical criteria established by Petersen et al. (2018). The threshold for memory impairment was derived from the same literature and designated as scoring >1.5 SD below the mean of age- and education-adjusted norms on a memory test. NC exclusion criteria included cognitive complaints and neurological or psychiatric disorders.

The study received ethical approval from the Institutional Review Committee of Nanjing Drum Tower Hospital (No. 2024-050-01), and all patients provided written informed consent.

### Cognitive and olfactory behavior measurements

All participants underwent a comprehensive neuropsychological assessment covering three cognitive domains: language [Animal Fluency Test (AFT) and Boston Naming Test (BNT)], episodic memory [Auditory Verbal Learning Test (AVLT) long-delayed memory and recognition], and executive function [Trail Making Test Part A (TMT-A) and Part B (TMT-B)]. Additionally, global cognition was assessed with the Mini-Mental State Examination (MMSE), Montreal Cognitive Assessment (MoCA), visuospatial ability with the Clock Drawing Test (CDT) and Rey-Osterrieth Complex Figure (ROCF) copy test, visuospatial memory with the ROCF recall test, and attention performance with the Symbol Digit Modalities Test (SDMT).

The olfactory behavior test was conducted using a computerized olfactory function evaluation instrument (OLFACTTM, Osmic Enterprises, Inc.^1^) outside the MRI scanner. The test assessed three aspects of olfactory behavior: threshold, identification, and memory. (1) Threshold Test (score range 1–13.5, using 14 channels of the olfactometer): participants were presented with varying concentrations of n-butanol and asked to identify the higher concentration. Correct identification led to a decreased concentration difference in subsequent trials, while incorrect identification increased the concentration difference. The olfactory threshold was calculated based on the average of the last two inversion points after three repetitions. Scores of 8–10 indicated normal olfactory sensitivity, 1–3 indicated olfactory dysfunction or anosmia, and scores ≥10 indicated better olfactory sensitivity. (2) Identification and Memory Test (using 20 channels of the olfactometer): this test was divided into two parts. In Task A (score range 0–10), participants were presented with 10 different odors and asked to identify each odor from four picture options. After a 10-min rest, Task B (score range 0–20) involved 20 odors, including the 10 odors from Task A and 10 new odors. Participants identified each odor from a set of four picture options and determined whether it was old or new.

### MRI acquisition

The fMRI data was acquired using a Philips Ingenia CX 3.0T MR with a 32-channel head coil. A T2*-weighted EPI sequence sensitive to blood oxygen level dependent (BOLD) signals was employed with the following parameters: repetition time (TR) = 2,000 ms, echo time (TE) = 30 ms, flip angle (FA) = 90°, field of view (FOV) = 192 mm × 192 mm, voxel size = 3 mm × 3 mm × 4 mm, 35 axial slices with a slice thickness of 4 mm, number of repetitions = 222, and a total acquisition time of 7 min and 24 s. T1-weighted images (T1WI) were acquired with these parameters: 196 sagittal slices, TR = 8.10 ms, TE = 3.70 ms, FOV = 256 mm × 256 mm, voxel size = 1 mm × 1 mm × 1 mm, and slice thickness of 1 mm.

### Odor-induced fMRI paradigm

We employed a standard event-related olfactory stimulation paradigm using lavender fragrance, as previously described (Zhang et al., 2018; Zhang et al., 2024), chosen for its minimal trigeminal stimulation, as the stimulus odorant (Givaudan Flavors Corporation, East Hanover, NJ, USA) in the MRI scanner. The paradigm included alternating conditions of “smell + odor” and “smell.” In the “smell + odor” condition, lavender was released to subjects with visual cues of “smell.” In the “smell” condition, odorless fresh air was presented with the same visual cues. An MRI-compatible olfactometer (Emerging Tech Trans, LLC, Hershey, PA, USA) delivered the odor to the subject’s nostrils, and visual cues were provided through the fMRI visual and audio stimulation system (Shenzhen Sinorad Medical Electronics Co., Ltd.) during the fMRI scanning. Lavender at four concentrations (0.032%, 0.10%, 0.32%, and 1.0% diluted in 1,2-propanediol, Sigma, St. Louis, MO, USA) was used sequentially to offset habituation effects, with each intensity presented three times before increasing to the next level.

### Imaging preprocessing

The preprocessing of olfactory task fMRI data was conducted using the DPABI software package based on Statistical Parametric Mapping 12 (SPM12) and included the following steps: (1) Converting T1 and fMRI raw data from DICOM to NIFTI format. (2) Excluding the first six time points of each scan to remove initial signal fluctuations. (3) Correcting for head movement and eliminating subjects with translation >3 mm and/or rotation >3°. (4) Coregistering T1-weighted high-resolution anatomical images to the mean functional image, segmenting them using a unified segmentation algorithm, and normalizing them to the Montreal Neurological Institute space template with a resolution of 1 mm × 1 mm × 1 mm. Functional images were normalized using the same parameters with a resolution of 3 mm × 3 mm × 3 mm. (4) Smoothing all images with a Gaussian kernel (FWHM 8 mm × 8 mm × 8 mm). (4) Assessing low-frequency (0.01–0.08 Hz) fluctuations in task fMRI signals to reflect spontaneous neuronal activity.

At the individual level, a high-pass filter synchronized the time series of the stimulus paradigm with each participant’s functional brain imaging data. The data was then modeled using a block design General Linear Model (GLM). The time series for both “smell + odor” and “smell” conditions were incorporated as predictor variables, with the Hemodynamic Response Function (HRF) convolved with the stimulus paradigm to correlate brain activity with the stimuli. This process yielded statistical parameter maps for each participant, reflecting neural responses under both conditions. Any subtle motion related to the paradigm was accounted for by adjusting head motion parameters as covariates.

### Generalized psychophysiological interaction analysis

The functional seed-to-voxel analysis was performed using the generalized psychophysiological interaction (gPPI) method from the SPM toolbox (McLaren et al., 2012). This task-based FC study identifies voxels or regions of interest (ROIs) with BOLD response patterns correlated with seed regions under specific psychological contexts. Unlike traditional psychophysiological interaction (PPI), gPPI uses task regressors to minimize co-activation effects. The steps were: (1) Defining the bilateral primary olfactory cortex (bPOC) as the ROI, based on prior literature (Lu et al., 2018). (2) Calculating the bPOC time series for each participant. (3) Deconvolving the time series with the HRF to obtain a new series representing physiological variables (smell + odor > smell). (4) Multiplying the step 2 time series by block onset times (“smell + odor”) and reconvolving with the HRF to get the PPI. (5) Creating a model for each participant with psychological variables (contrast “smell + odor > smell”), physiological variables, and PPI to map connectivity between the bPOC and other brain regions during odor stimulation. (6) Identifying brain regions with significant differences between the bPOC and the whole brain as new ROIs, followed by a whole brain gPPI analysis.

### Brain activation analysis

In this study, we focused on the “smell + odor > smell” condition. To visualize the bPOC activation patterns for all subjects, olfactory activation maps were generated using one-sample *t*-tests [*p* < 0.005, corrected for Gaussian Random Field (GRF)]. Then we extracted the average activation values of bPOC for each subject from the activation maps using the DPABI software package. Minor movements correlated with the paradigm were corrected by incorporating movement parameters as covariates during the paradigm estimation step.

### Statistical analysis

Clinical data analyses were conducted using SPSS version 24.0 (SPSS Inc., Chicago, IL, USA). Demographic information, clinical variables, and cognitive and olfactory behavior assessment scores were reported as mean ± SD and compared between groups using two-sample *t*-tests or Mann–Whitney *U* tests, depending on whether the data followed a normal distribution. Chi-square tests (χ^2^) were used for categorical data. Cognitive test scores were converted to *z*-scores using Fisher’s z-transformation method. For cognitive domains with multiple tests, the *z*-scores were averaged.

To assess differences in odor-induced bPOC activity and FC of olfactory neural circuits between groups, a two-sample *t*-test was performed, with *p* values corrected for GRF at a voxel-level threshold of *p* < 0.005 and a cluster-level threshold of *p* < 0.05 using DPABI software. For brain regions showing significant between-group differences, the bPOC activity values and the FC values were extracted for subsequent correlation analyses. Spearman correlation analyses were conducted to examine the associations between the FC of neural circuits and cognitive function as well as olfactory behavior scores. A *p*-value of less than 0.05 was considered statistically significant.

## Results

### Demographic, cognition status, and olfactory behavior

There were no significant differences in age, gender, and education between MCI and NC subjects. Patients with MCI had lower general cognition status in MMSE and MoCA, episodic memory, visuospatial memory, executive function, language ability, and attention performance compared with NC subjects. No significant differences were observed between the two groups in the olfactory identification and olfactory memory ability; however, MCI patients demonstrated a lower olfactory threshold and total olfactory ability using a computerized olfactory function evaluation instrument outside the MRI scanner (Table 1).

**TABLE 1:** Demographic, cognition status, and olfactory behavior.

	NC (*n* = 42)	MCI (*n* = 39)	Statistics	*p*
Age (years)	65.57 ± 4.29	65.82 ± 6.64	−0.047^a^	0.962
Gender (M/F)	13/29	16/23	0.345^b^	0.365
Education (years)	12.00 ± 2.29	11.72 ± 3.47	−0.719^a^	0.472
MMSE	28.71 ± 0.99	27.95 ± 1.75	2.400^c^	0.020
MoCA	25.57 ± 1.74	23.23 ± 2.97	−3.704^a^	**<0.001**
**Episodic memory**				
AVLT immediate	16.81 ± 4.11	12.54 ± 3.43	−4.473^a^	**<0.001**
AVLT short-delayed	5.48 ± 2.01	3.03 ± 1.97	−4.880^a^	**<0.001**
AVLT long-delayed	4.76 ± 2.14	2.56 ± 1.92	−4.449^a^	**<0.001**
AVLT cued recall	4.83 ± 2.06	2.05 ± 1.72	−5.417^a^	**<0.001**
AVLT recognition	21.19 ± 3.26	18.54 ± 2.60	−5.318^a^	**<0.001**
*z*-Scores	0.48 ± 0.67	−0.52 ± 0.65	−5.633^a^	**<0.001**
**Visuospatial memory**				
ROCF recall	15.60 ± 6.83	9.53 ± 7.25	3.880^c^	**<0.001**
*z*-Scores	0.38 ± 0.89	−0.41 ± 0.95	3.880^c^	**<0.001**
**Executive function**				
TMT-A, s	55.12 ± 15.25	83.67 ± 35.20	−4.340^a^	**<0.001**
TMT-B, s	142.48 ± 29.44	182.59 ± 71.48	−2.170^a^	**0.030**
*z*-Scores	−0.39 ± 0.42	0.43 ± 1.13	−3.469^a^	**0.001**
**Language ability**				
BNT	26.71 ± 2.59	24.38 ± 3.46	−3.090^a^	**0.002**
AFT	19.10 ± 5.61	15.90 ± 4.42	−2.614^a^	**0.009**
*z*-Scores	0.32 ± 0.83	−0.34 ± 1.06	−3.162^a^	**0.002**
**Visuospatial ability**				
ROCF copy	34.33 ± 3.65	33.36 ± 5.14	−0.757^a^	0.449
CDT	26.40 ± 3.51	24.33 ± 5.66	1.994^c^	0.050
*z*-Scores	0.16 ± 0.55	−0.17 ± 0.84	−1.867^a^	0.062
**Attention performance**				
SDMT	38.05 ± 7.49	32.18 ± 9.41	3.115^c^	**0.003**
*z*-Scores	0.32 ± 0.84	−0.34 ± 1.06	3.115^c^	**0.003**
**Olfactory function**				
Olfactory threshold	7.61 ± 3.68	5.33 ± 3.46	−3.093^a^	**0.002**
*z*-Scores	0.29 ± 0.98	−0.32 ± 0.93	−3.093^a^	**0.002**
**Olfactory identification**				
Task A (10 odors)	7.36 ± 1.65	6.67 ± 1.91	−1.613^a^	0.107
Task B (20 odors)	12.29 ± 3.37	11.87 ± 2.98	−1.017^a^	0.309
*z*-Scores	0.12 ± 0.89	−0.13 ± 0.89	−1.522^a^	0.128
**Olfactory memory**				
Old 10 odors	7.48 ± 2.13	7.26 ± 2.54	−0.101^a^	0.920
New 10 odors	6.31 ± 2.16	5.90 ± 2.25	−0.878^a^	0.380
*z*-Scores	0.07 ± 0.70	−0.07 ± 0.66	−1.414^a^	0.157
Total scores	0.13 ± 0.61	−0.13 ± 0.62	−2.259^a^	**0.024**

Data are mean ± SD.

“a” represents Mann–Whitney *U*-test;

“b” represents Pearson Chi-square test;

“c” represents Independent Samples *t*-test. Olfactory identification task has 20 odors; olfactory memory task includes the old 10 odors in olfactory identification task. NC, normal control; MCI, mild cognitive impairment; MMSE, Mini-Mental State Examination; MoCA, Montreal Cognitive Assessment, Beijing Version; AVLT, Auditory Verbal Learning Test; ROCF, Reye-Osterrieth Complex Figure; TMT-A, Trail Making Test Part A; TMT-B, Trail Making Test Part B; AFT, Animal Fluency Test; BNT, Boston Naming Test; CDT, Clock Drawing Test; SDMT, Symbol Digit Modalities Test. *P* < 0.05 are indicated in bold font.

### Alterations in odor-induced bPOC activation: NC and MCI

**FIGURE 1:**
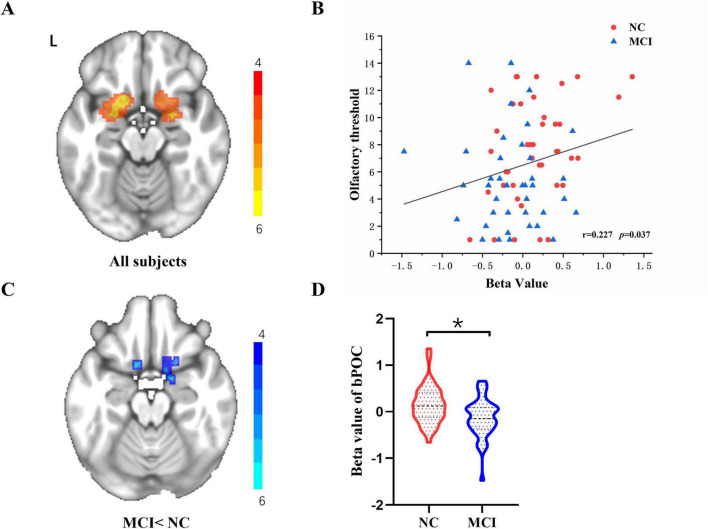
**(A)** One-sample *t*-test across all 81 subjects for the odor-induced bPOC activation. **(B)** Increased odor-induced bPOC activity was associated with higher olfactory threshold scores in the whole cohort, as determined by Spearman correlation analysis. *p < 0.05 was considered significant. **(C,D)** Comparison of the beta values of odor-induced bPOC activation between NC and MCI patients (with GRF correction, voxel *p* < 0.005, cluster *p* < 0.05).

Patients with MCI and NC illustrated extensive bPOC activation under odor stimulation during fMRI scanning (Figure 1A). In the whole cohort, a significant positive correlation between the activation beta values of bPOC and the olfactory threshold was found (Figure 1B). Compared to the NC, MCI patients exhibited significantly decreased bPOC activation under odor stimulation (GRF correction, voxel *p* < 0.005, cluster *p* < 0.05) (Figures 1C, D).

### Odor-induced functional connectivity alterations in MCI

**FIGURE 2:**
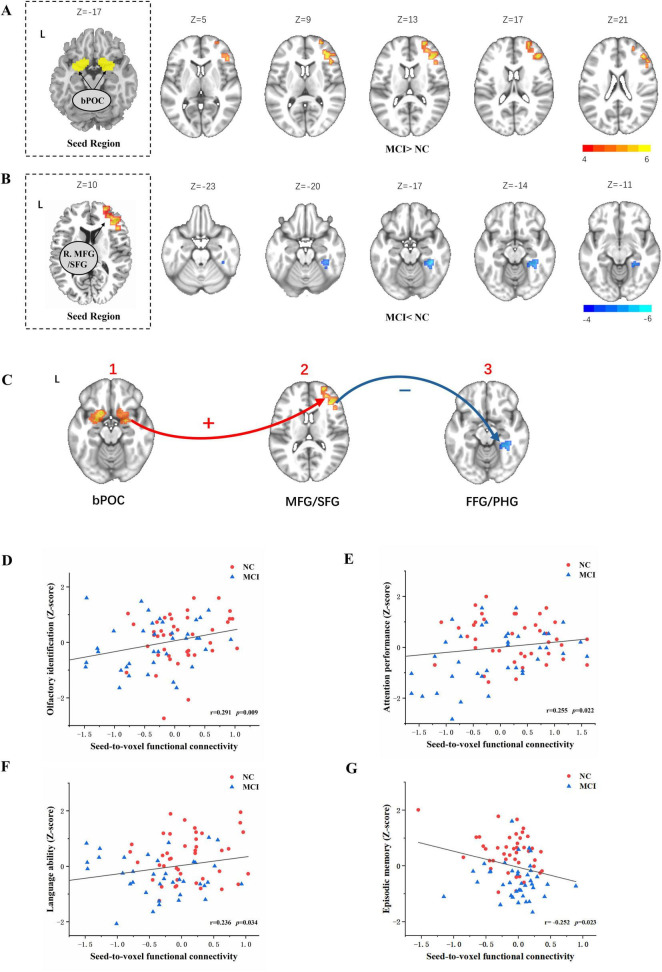
Odor-induced seed-to-voxel functional connectivity alteration for the bPOC and right MFG/SFG as ROIs and its relationship with olfactory behavior tests and cognitive performance. Independent-sample *t*-test corrected for age, education, and gender indicated significantly enhanced brain functional connectivity (bPOC to the right MFG/SFG) in the subjects with MCI compared with NC during odor stimulation (with GRF correction, voxel *p* < 0.001, cluster *p* < 0.05) **(A)**. A significantly decreased connectivity from the right MFG/SFG to the right FFG/PHG during odor stimulation was found in MCI subjects corrected for age, education, and gender (with GRF correction, voxel *p* < 0.001, cluster *p* < 0.05) **(B)**. The odor-induced FC changes in the POC-frontal cortex–medial temporal cortex circuit in MCI subjects compared to NC subjects **(C)**. Decreased functional connectivity from the right SFG/MFG to the right FFG/PHG was positively associated with *z*-scores of cognitive domains including language ability, attention performance, and olfactory identification tests, as determined by Spearman correlation analysis **(D–F)**. Increased functional connectivity from bPOC to the right SFG/MFG were negatively associated with *z*-scores of episodic memories **(G)**. bPOC, bilateral primary olfactory cortex; FFG, fusiform gyrus; PHG, parahippocampal gyrus; MFG, middle frontal gyrus; SFG, superior frontal gyrus; MNI, Montreal Neurological Institute; L, left; R, right.

Mild cognitive impairment patients showed increased FC during odor induced fMRI scanning from the bPOC to the anterior frontal lobe [including the right middle frontal gyrus (MFG) and superior frontal gyrus (SFG)] in gPPI analysis using the bPOC as ROI (GRF correction, voxel *p* < 0.005, cluster *p* < 0.05) (Table 2 and Figure 2A) compared with NC.

**TABLE 2:** Brain regions with significant inter-group differences in functional connectivity with the bPOC under olfactory stimulation.

Brain regions	Cluster size	Peak MNI			*t*
		*X*	*Y*	*Z*	
MCI > NC					
R. MFG	156	45	36	18	4.3397
R. SFG					

Independent-sample *t*-test on inter-group differences in functional connectivity with the bPOC under olfactory stimulation in patients with MCI and NC (GRF correction, voxel *p* < 0.005, cluster *p* < 0.05). bPOC, bilateral primary olfactory cortex; MFG, middle frontal gyrus; SFG, superior frontal gyrus; MNI, Montreal Neurological Institute; R, right.

To gain deeper insights into the potential alterations in interactions between the anterior frontal lobe and other brain regions when the connectivity from the bPOC to the right SFG/MFG is altered in MCI patients, and to better comprehend the pivotal role of the right anterior frontal lobe in odor processing among MCI subjects, we performed a gPPI analysis with the right SFG/MFG as the ROI. Compared to NC subjects, decreased FC from the right SFG/MFG to the right medial temporal cortex [including the right fusiform gyrus (FFG) and parahippocampal gyrus (PHG)] was found in patients with MCI (GRF correction, voxel *p* < 0.005, cluster *p* < 0.05) (Table 3 and Figure 2B).

**TABLE 3:** Brain regions with significant inter-group differences in functional connectivity with the right MFG/SFG under olfactory stimulation.

Brain regions	Cluster size	Peak MNI			*t*
		*X*	*Y*	*Z*	
MCI < NC					
R. FFG	33	36	−36	−18	−3.7375
R. PHG					

Independent-sample *t*-test on inter-group differences in functional connectivity with the right superior and middle frontal gyrus under olfactory stimulation in patients with MCI and NC (GRF correction, voxel *p* < 0.005, cluster *p* < 0.05). FFG, fusiform gyrus; PHG, parahippocampal gyrus; MFG, middle frontal gyrus; SFG, superior frontal gyrus; MNI, Montreal Neurological Institute; R, right.

The odor-induced FC changes were primarily observed in the POC-anterior frontal cortex-medial temporal cortex circuit in MCI subjects, compared to NC subjects (Figure 2C).

### Associations of altered functional connectivity with neuropsychological test scores and olfactory behavior

To further investigate the relationship between these FC patterns in the olfactory neural circuit and olfactory behavioral performance, and to uncover potential neural mechanisms underlying olfactory behavior in the MCI population, we performed Spearman correlation analyses for both groups. Increased FC from the bPOC to the right SFG/MFG was negatively associated with *z*-scores of episodic memory (*r* = −0.252, *p* = 0.023) (Figure 2G). Decreased FC from the right SFG/MFG to the right FFG/PHG was positively associated with *z*-scores of attention performance (*r* = 0.255, *p* = 0.022), language ability (*r* = 0.236, *p* = 0.034), and olfactory identification (*r* = 0.291, *p* = 0.009) (Figures 2D–F).

## Discussion

This study demonstrated odor-induced FC changes in the POC-anterior frontal cortex-medial temporal cortex circuit in MCI subjects. We observed significantly impaired bPOC activation under odor stimulation, suggesting that primary central olfactory structures, beyond the OB and tract, are involved in the pathology of AD. Importantly, the association between the increased FC from bPOC to the right anterior frontal lobe and episodic memory may represent a compensatory mechanism in MCI patients, helping them maintain normal olfactory and cognitive function. Decreased FC from the right anterior frontal lobe to the right medial temporal cortex in patients with MCI was positively associated with attention performance, language ability and olfactory identification ability, indicating that compensatory mechanisms have already failed at this stage. This suggests that early sensitive changes in cognitive decline are characterized by a reduction in the coordination of information transmission between key olfactory brain regions, which occurs prior to the decline in the functionality of individual brain regions in MCI patients. This alteration in coordination is predominantly observed in the primary olfactory cortex.

The observed increase in FC from the bPOC to the right anterior frontal lobe in MCI patients contrasts with previous research. Typically, the FC between the olfactory system and the medial prefrontal cortex in rodent models provides a neural basis for the olfactory modulation of cognition, with the flow of communication appearing to proceed from the perirhinal cortex to the medial prefrontal cortex (Bhattarai et al., 2022; Hopkins et al., 2023). Our findings suggest a compensatory mechanism where the bPOC may attempt to engage more frontal resources to process olfactory information despite the ongoing degeneration. This increased connectivity might reflect an attempt by the brain to maintain cognitive function through enhanced engagement of higher-order processing areas. However, the decreased FC from the right anterior frontal lobe to the right medial temporal cortex aligns with prior research indicating that MCI is associated with disruptions in medial temporal lobe connectivity. Although the literature suggests that the posterior medial temporal lobe network is particularly vulnerable to early AD, Das et al. (2015) demonstrated that both the anterior and posterior medial temporal lobe networks are affected in MCI compared to NC. Patients with AD showed decreased fALFF in the right inferior temporal gyrus and lower olfactory function was associated with smaller volumes in the hippocampus, parahippocampal gyrus and fusiform gyrus (Bothwell et al., 2023; Hari et al., 2023). Studies have reported that accelerated atrophy of the medial temporal lobe and parahippocampal gyrus is associated with an increased risk of developing AD (Papadatos and Phillips, 2023). The medial temporal lobe, crucial for memory, and olfactory processing, relies on efficient communication with frontal regions to integrate and interpret sensory information (van Staalduinen and Zeineh, 2022). Previous study found that reduced medial temporal-cortical connectivity in MCI patients was related to reduced entorhinal thickness and white matter integrity of the parahippocampal (Berron et al., 2020). Reduced FC in this pathway suggests impaired integration and memory consolidation processes in MCI, which could contribute to the cognitive deficits observed.

The POC-anterior frontal cortex-medial temporal cortex circuit is essential for integrating sensory inputs and memory processes. Our study reveals significant alterations in this circuit in MCI patients. Specifically, the increased FC from the bPOC to the anterior frontal cortex, coupled with decreased connectivity to the medial temporal cortex, suggests that while the initial sensory processing may remain relatively intact or even enhanced, the subsequent integration and memory processes are impaired. Prior research have indicated a trend toward reduced volume of the POC in patients with AD or MCI compared to healthy controls (Jobin et al., 2021a). This suggests that neuroanatomical structures involved in olfactory processing are smaller in AD, and such volumetric reductions may be detectable as early as the MCI stage. A meta-analysis conducted using computer software analysis revealed that alterations in the default mode network in patients with MCI primarily involve the bilateral frontal lobes, bilateral temporal lobes, and the PHG (Yuan et al., 2021). Previous studies also have shown that the POC is crucial for initial olfactory processing, while the frontal and temporal cortices are involved in higher-order processing and memory integration (Daulatzai, 2015). The mesial temporal lobes interact with the frontal lobes in various aspects of memory, including learning, recognition, and memory errors, in AD (Keith et al., 2023). The medial temporal lobe and prefrontal cortex are heavily interconnected and do not operate independently in underlying working memory and olfactory association (Wang et al., 2020; Wu and Buckley, 2022). The disruption observed in this study supports the hypothesis that MCI affects the coordination within this circuit, leading to inefficient processing and integration of olfactory and cognitive information. These findings are consistent with the broader literature indicating that MCI impacts neural circuits involved in multisensory processing and memory.

Recent studies have indicated that individuals with MCI exhibit reduced olfactory identification, with this impairment worsening as the disease progresses within the AD spectrum, potentially aiding in the prediction of cognitive decline (Guo et al., 2023; Touliou et al., 2021). Unlike previous studies, our research found that MCI patients have decreased olfactory thresholds but normal olfactory identification. This may be because early MCI primarily disrupts basic olfactory perception (like thresholds) rather than more complex functions such as identification and memory. It is also possible that in this study, the concentration of odor compounds used for olfactory identification was too high, which may have caused the impaired threshold levels not to affect the olfactory identification function in MCI patients. The reduced activation of the bPOC underscores the neural basis of olfactory deficits in patients with MCI. Throughout the cohort, the bPOC exhibited substantial activation in response to odor stimulation. Furthermore, the activation beta values of the bPOC showed a significant positive correlation with the olfactory threshold, aligning with previous research findings, which indicate that decreased olfactory cortical activation is associated with reduced sensitivity to odors (Karunanayaka et al., 2014; Karunanayaka et al., 2017). This supports the notion that early olfactory dysfunction in MCI may be rooted in decreased cortical activation and could potentially serve as an early biomarker for detecting the disease.

The correlations between odor induced FC patterns and neuropsychological test scores provide additional insights into the relationship between olfactory processing and cognitive performance in MCI. The negative correlation between increased FC from the bPOC to the right SFG/MFG and episodic memory *z*-scores suggests that while increased connectivity might reflect compensatory mechanisms, it does not necessarily enhance cognitive performance. This may imply that such compensatory connectivity is insufficient to fully counteract the cognitive decline associated with MCI. Conversely, the positive correlations between decreased FC from the right SFG/MFG to the right FFG/PHG and better performance in attention, language ability, and olfactory identification are intriguing. They suggest that more efficient connectivity in this pathway may be associated with preserved or even enhanced cognitive and olfactory functions in MCI patients. This highlights the complexity of FC changes and their differential impacts on various cognitive and sensory domains.

This study has several limitations that should be considered. Firstly, the cross-sectional design limits our ability to infer causal relationships or track changes over time. Longitudinal studies are needed to determine how these connectivity alterations evolve and their prognostic value for MCI progression. Secondly, the relatively small sample size in this study reduces the reliability of the results. According to the simulation-based approach described by Desmond and Glover (2002), achieving 80% statistical power at a voxel-wise threshold of α = 0.05 requires a minimum of 12 participants in an fMRI study. For stricter thresholds, such as those adjusted for multiple comparisons, it is recommended to double the sample size to maintain this level of power. So, it remains crucial to validate these findings in larger, more diverse populations to enhance generalizability. Thirdly, the olfactory threshold test was conducted using n-butanol, and the stimulus gas for the olfactory task fMRI scans was lavender. In future studies, we could explore the sensitivity of the olfactory threshold test to different concentrations of lavender. Additionally, exploring other sensory modalities and their interactions with cognitive functions could provide a more comprehensive understanding of sensory-cognitive integration in MCI.

## Conclusion

In summary, this study demonstrates that MCI is associated with significant alterations in the FC of the POC-anterior frontal cortex-medial temporal cortex circuit. The findings suggest that the primary site of dysfunction in MCI is the POC, which shows reduced activation and altered connectivity patterns. These changes in connectivity are linked to both olfactory behavior and cognitive performance, indicating that disruptions in olfactory neural circuits may serve as an early marker for MCI. Future research should aim to elucidate the temporal dynamics of these changes and their potential as targets for early intervention strategies.

## Data Availability

The original contributions presented in this study are included in this article/supplementary material, further inquiries can be directed to the corresponding authors.

## Footnotes

1
http://www.osmicenterprises.com/
